# Central Nervous System Responses to Simulated Galactic Cosmic Rays

**DOI:** 10.3390/ijms19113669

**Published:** 2018-11-20

**Authors:** Egle Cekanaviciute, Susanna Rosi, Sylvain V. Costes

**Affiliations:** 1 Universities Space Research Association (USRA), Moffett Field, CA 94035, USA; 2 Space Biosciences Division, NASA Ames Research Center, Moffett Field, CA 94035, USA; sylvain.v.costes@nasa.gov; 3 Department of Physical Therapy and Rehabilitation Science, University of California, San Francisco, CA 94158, USA; susanna.rosi@ucsf.edu; 4 Brain and Spinal Injury Center, University of California, San Francisco, CA 94143, USA; 5 Department of Neurological Surgery, University of California, San Francisco, CA 94143, USA; 6 Weill Institute for Neuroscience, University of California San Francisco, San Francisco, CA 94143, USA; 7 Kavli Institute of Fundamental Neuroscience, University of California San Francisco, San Francisco, CA 94158, USA

**Keywords:** central nervous system, radiation, spaceflight

## Abstract

In preparation for lunar and Mars missions it is essential to consider the challenges to human health that are posed by long-duration deep space habitation via multiple stressors, including ionizing radiation, gravitational changes during flight and in orbit, other aspects of the space environment such as high level of carbon dioxide, and psychological stress from confined environment and social isolation. It remains unclear how these stressors individually or in combination impact the central nervous system (CNS), presenting potential obstacles for astronauts engaged in deep space travel. Although human spaceflight research only within the last decade has started to include the effects of radiation transmitted by galactic cosmic rays to the CNS, radiation is currently considered to be one of the main stressors for prolonged spaceflight and deep space exploration. Here we will review the current knowledge of CNS damage caused by simulated space radiation with an emphasis on neuronal and glial responses along with cognitive functions. Furthermore, we will present novel experimental approaches to integrate the knowledge into more comprehensive studies, including multiple stressors at once and potential translation to human functions. Finally, we will discuss the need for developing biomarkers as predictors for cognitive decline and therapeutic countermeasures to prevent CNS damage and the loss of cognitive abilities.

## 1. Introduction

Astronauts on the International Space Station (ISS) are somewhat protected from galactic cosmic rays (GCRs) by the magnetic field of the Earth. However, deep space missions, including lunar and Mars orbit and surface, are estimated to lead to prolonged GCR exposure at rates up to 10-fold higher than on the ISS, which would amount to cumulative doses of 1–1.2 Sv over a three year-long mission to Mars [1]. The GCR spectrum is composed of 90% protons, 9% He ions and 1% heavier ions which are referred to as HZE particles (i.e., particles with high charge Z and high energy E) with energies ranging mainly between 0.1 and 1 GeV/n [2]. HZE particles typically have a linear energy transfer (LET) that is orders of magnitude higher than ionizing radiation measured on Earth such as X-rays or gamma rays. X-rays and gamma rays are known to deposit energy uniformly in the tissue, therefore are referred to as sparsely ionizing or low-LET radiation. In contrast, HZE particles typically deposit part of their energy along linear tracks referred to as cores, while the remaining energy is scattered randomly by energetic electrons (i.e., delta rays) outside the core, defining a region called the penumbra, thus HZE dosimetry can be approximated by a radial dose distribution decreasing as the distance square from the track [3,4]. HZE are often referred as densely ionizing or high-LET radiation as most of the energy is deposited only within a few µm from the core, while the penumbra is typically low dose (<0.01 Gy) deposited by sparsely ionizing electrons.

It has been a challenge to predict the response of a given tissue to HZE by extrapolating the more abundant low-LET data. The primary reason for this difficulty is that in contrast to low-LET where low dose implies a uniform reduction of ionizing radiation in all cells and tissues, a low dose exposure to HZE only means less tissue being traversed by particles, but each particle still producing the same amount of damage along its track. To predict HZE response inside a tissue, cell-cell and cell-ECM (extracellular matrix) communication becomes a critical component to consider. Such communication is typically triggered above a certain dose threshold, leading to non-linear dose response and unpredictable amplification of the impact of ionizing radiation to unexposed tissue which together have been called non-targeted effects [5]. The brain is a complex tissue and its spatial architecture with very specific orientation pattern may lead to very distinct responses depending on the characteristics of particle energy, charge and orientation. It is therefore difficult to imagine that in order to evaluate the risk from HZE to the central nervous system (CNS), how low-LET data alone would be sufficient. Instead, it is necessary to study the CNS response directly to HZE, and novel techniques will be required to distinguish non-targeted effects from targeted-effects. Beam orientation and a deeper understanding of the brain structure to the level of cell-cell interactions and intercellular differences of gene expression would probably be another parameter to track in experiments conducted in an accelerator simulating HZE.

It has been calculated that an individual cell in an astronaut will be traversed by protons once every three days, by helium nuclei once every three weeks, and by more than two nuclei of higher atomic number once every three months, which on an estimated three-year Mars mission could result in extensive exposure risks. The same estimates suggest that, during a Mars mission, more than 13% of total neurons may be directly affected by high atomic number space radiation particles [6]. However, high-LET radiation has been investigated less thoroughly than low-LET, in part due to experimental limitations: the lack of deep space missions with biological samples means that the results of GCR effects on the CNS are acquired in ground simulations either using single particles (protons, ^4^He, ^56^Fe, etc.) or mixed beams. In addition, it may be speculated that if each individual track can generate lasting CNS effects, then HZE exposure could lead to additive CNS effects, independently of the dose rate. This is in contrast with low-LET impact, where chronic doses lead to undetectable effects and thus there is little rationale to infer additive effects for the CNS. It is therefore critical to better understand composite effects of multiple ion radiation that astronauts will face during deep space travel in order to accurately predict the risk of CNS damage.

## 2. The Effects of HZE Particle Irradiation on Behavioral and Cognitive Functions in Rodents

As stated in the previous section, to ensure safety of personnel and mission success during deep space travel it is critical to understand the consequences of GCRs upon various ethologically relevant cognitive and behavioral domains. Recent studies have begun to characterize a number of maladaptive behavioral responses associated with individual ion exposures in rodents. The maladaptive cognitive and behavioral changes are characterized by alterations in learning and memory, fear/startle responses, anxiety phenotypes and social behaviors with responses varying depending on both the dose and type of ion exposure.

**Figure 1:**
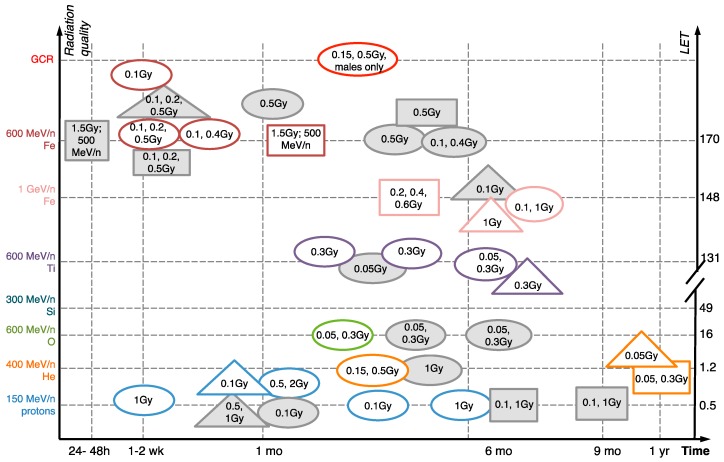
Development of memory impairments after HZE ion and simulated GCR irradiation [6,8,9,10,11,12,13,14,15,16,17,18,19,20,21,22]. Ovals, novel object recognition. Squares, spatial memory (Morris water maze, Barnes maze, Radial Arm maze). Triangles, fear conditioning. Doses (Gy) listed inside the shapes. Grey color, negative data (showing no impairment). White, positive. All findings are in males unless noted otherwise. All studies done on mice except [18].

Cognitive and behavioral functions in rodents can be evaluated based on tasks similar to those used for humans. Here we provide a few examples of the commonly used tests to represent the wide range of cognition aspects and behaviors that are altered by simulated space radiation: Recognition memory, social memory, spatial memory, anxiety and attention. For instance, human recognition memory is defined as the ability to identify a previously encountered object and/or situation as familiar. Rodent recognition memory can be reliably tested using the Novel Object Recognition test (NOR) [7]. This behavioral task measures recognition memory without the use of aversive stimuli (shocks, water, noise) and is based on the natural tendency of rodents to explore novel situations and objects. The NOR (also known as the “visual paired-comparison task” in studies on humans and monkeys) is a highly used and highly reproducible metric for quantifying the recognition memory formed by the hippocampus, which is one of the brain regions required for the formation of new memories and regulating social memories. Another similar test to NOR is object-in-place, which compares the time spent with two objects, one of which remains in the same location and the other changes locations [8]. These tests are particularly sensitive to radiation: Multiple studies have demonstrated NOR deficiencies induced by as little as 0.1 Gy of 600 MeV/n ^56^Fe within 1 week of exposure [9,10,11], while deficiencies in spatial memory and fear conditioning occur between 1 month-1 year after irradiation (NOR and other cognitive impairments following irradiation are summarized in Figure 1). 

Social memory is essential for engaging in appropriate behaviors and meaningful relationships in socially behaving species like humans and mice. Understanding how the harsh environment of deep space will impact astronaut cognitive function, including social behaviors, is critical for achieving mission success. Social functions in rodents are studied by measuring natural propensity for social interaction as well as social novelty for spending time with a previously un-encountered cage-mate: Normal social interaction involves significantly longer time spent with previously un-encountered rodent compared to a rodent that had been encountered before. Two recent reports have identified long-term deficits in social memory following exposure to 0.5 Gy GCR and 0.25 Gy 600 MeV/n ^16^O in male mice [6,22]. Similarly, 0.05 Gy and 0.25 Gy 1000 MeV ^16^O exposure has been shown to inhibit social odor recognition memory in male rats [23].

Anxiety, which in humans is a state of fearfulness, concern or apprehension, can be measured in rodents by an aversion to enter and/or remain in open, often brightly lit areas using the elevated plus maze [24]. Anxiety-like phenotypes have been reported in rodents chronically after GCR exposure [6] and up to one-year post helium exposure alone [20] suggesting that these disorders are persistent when induced by radiation.

Spatial working memory is also regulated by the hippocampus and can be tested using the Barnes maze, which is measured by the time it takes for the rodent to find the escape box based on environmental cues around the maze (“escape latency”). Exposure to 0.2–0.6 Gy of 1 GeV/n ^56^Fe particles is sufficient to significantly prolong Barnes maze escape latency in rats 3 months after irradiation, indicating impairment in working spatial memory. Notably, significantly lower doses of ^56^Fe are required for this effect compared to X-rays (8–13 Gy of 125 kVp X-rays) [18]. 

In addition to multiple aspects of memory formation and retrieval, HZE ions also alter prefrontal cortex-dependent cognitive functions. Operant responding measures performance based on ascending fixed ratio of reward: In this test, rodents press a lever in expectation for reward, and as the rewards become more sparse, the animals have to increase the frequency of responses (lever presses) to obtain a reward. At low ratios (i.e., rewards being common), operant responding primarily measures the motivation to achieve the food reward, and at high ratios, when the rewards become more rare, it instead begins to measure the activational value of the reward and animal’s willingness to work for a given reinforcer. Operant responding is primarily regulated by the dopaminergic system in the brain. In rats, 1.5 Gy doses of ^56^Fe has been reported to dysregulate it starting at 7 months and continuing to up to 15 months after exposure [25,26], indicating that HZE ion exposure may alter the dopaminergic system. Another cognitive test that also relies on the functions of the dopaminergic system, the psychomotor vigilance test, is based on increasing the waiting time before response and measures premature responses that can reflect a drop in sustained attention and increased impulsivity. The psychomotor vigilance test has also shown an impairment in rats after proton irradiation that was correlated with the strength of dopaminergic signaling and helped distinguish between radiation-sensitive and radiation-resistant animals [27] and uncover a genetic/strain-dependent component to radiation sensitivity [28].

## 3. Individual HZE Ion and Combined GCR Effects on Neuronal Damage and Neuroinflammation in Rodents

**Figure 2:**
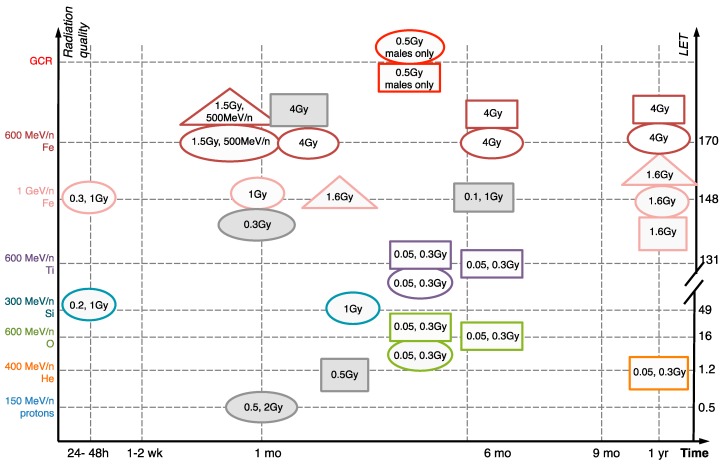
Development of neuronal damage and neuroinflammation after HZE ion and simulated GCR irradiation. [6,8,13,14,17,19,20,29,30,31,32] Ovals, dendrite/synapse/receptor/neuronal loss. Squares, gliosis (astrogliosis and/or microgliosis). Triangles, oxidative stress. Doses (Gy) listed inside the shapes. Grey color, negative data (i.e. showing no impairment). White, positive. All findings are in males unless noted otherwise. All studies done on mice except [32].

The cognitive and behavioral changes cited above have been associated with synapse loss and neuroinflammation, which have provided the initial cellular basis for the effects of single HZE ions as well as combined multi-ion GCRs in the nervous system (summarized in Figure 2 and in Table 1). Synapses are neuronal connections that transfer signals between neurons using chemical neurotransmitters, such as glutamate (excitatory) or gamma-amino-butyric acid (inhibitory). Synapses are commonly formed between two neuronal processes: An axon from the presynaptic neuron that is sending the signal and a dendrite of the receiving postsynaptic neuron, typically onto its protuberance called a dendritic spine.

HZE ions have been demonstrated to inhibit neuronal connectivity and synaptic activity, as evidenced by the loss of dendritic spines, dendrite length and branching 3 months after exposure to 0.05 Gy or 0.3 Gy of 600 MeV/n ^16^O or ^48^Ti [8]. Higher doses, such as 4 Gy of 600 MeV/n Fe, also cause axonal degeneration [32]. Synaptic and dendritic damage following proton irradiation is correlated with impaired electrophysiological functions in areas responsible for memory formation, for example, reduced excitability of hippocampal neurons in response to 1 Gy of 150 MeV/n protons [33].

Recently reported cognitive impairments and synapse alterations in response to HZE particles— specifically, 0.05–0.3 Gy of 600 MeV/n ^48^Ti, or ^16^O, or 0.15–0.5 Gy of combined GCR simulation— correspond with modified inflammatory response and enhanced microglia activation up to 12 months after exposure [6,8]. Microglia are the resident macrophages and the main immune component in the brain, accounting for 10–15% of all brain cells. They constantly survey for signals of injury or infection, quickly moving toward affected sites upon activation, acting as key mediators of neuroinflammatory processes. It has long been established that microglia can passively regulate neuronal health through the release of cytokines and chemokines, however, more recent reports found direct effects of microglia on synapse function that can be dependent on the complement cascade [34]. To this extent, a recent report has shown that after ^4^He exposure microglia express elevated levels of scavenger receptors, lysosome membrane proteins and complement receptors, which correlate with synapse loss [13]. The complement cascade forms part of the innate immune defense responses, but in the CNS has also been implicated in selective pruning of synapses during neuronal development and in neurological diseases. Specifically, complement proteins have been demonstrated to tag the synapses that are subsequently phagocytosed by microglia [34,35], and complement component C3 deficiency is neuroprotective and reduces neuroinflammation in a model of Alzheimer’s disease [36], and prevents cognitive decline during normal aging [37]. These studies suggest a potential mechanism that combines synapse loss and microglial activation during the development of cognitive deficits after HZE ion exposure.

Neuroinflammation mediated by CNS immune cells, microglia, could be exacerbated by systemic immune response to irradiation if the blood-brain barrier were compromised and allowed the influx of peripheral immune cells to the brain. However, the impact of HZE ions and simulated GCRs on the blood-brain barrier has been under-investigated: The majority of relevant research has primarily been focused on gamma instead of particle radiation [38]. Nonetheless, the hypothesized interaction between CNS and peripheral inflammation mediated by a leaky blood-brain barrier in response to HZE ions may account for the fact that whole body 1 GeV/n ^16^O irradiation has been shown to lead to worse behavioral impairments than head irradiation alone in rats [39], and for the fact that peripheral immune cells can serve as biomarkers for the severity of radiation-induced behavioral deficits in response to 0.25 Gy of 600 MeV/n ^16^O irradiation in mice [22].

HZE ion-induced neuronal damage has been shown to be directly associated with mitochondrial dysfunction and the production of reactive oxygen species (ROS) [40]. During the process of cellular respiration, mitochondria generate ROS such as hydrogen peroxide and superoxide as a byproduct. Ionizing radiation stimulates ROS production both by inducing mitochondrial damage and directly by water oxidation, and excessive levels of ROS in one cell can cause unrepairable damage and death, which could cascade to death of neighboring cells as well. ROS-mediated damage after ionizing radiation can be partially rescued by pharmacological or genetic reduction of oxidative stress [14,17,41]. ROS production may be particularly detrimental to neural precursor cells that are involved in hippocampal neurogenesis in both human and rodent CNS.

By analogy to CNS disease models, neuronal damage and neuroinflammation might be hypothesized to form a positive feedback loop in which signaling by apoptotic cells and ROS production activate brain microglia and peripheral macrophages, which are necessary for removing cellular debris, but can also further damage neurons by destroying synapses via the complement cascade [34]. ROS production and neuronal damage have indeed been shown by multiple studies to be accompanied by microglial activation [42].

## 4. Experimental and Epidemiological Variables That Influence HZE Ion Effects in the CNS 

The time course for the development of cognitive and behavioral deficits after HZE ion exposure varies widely between experiments. Some of the impairments, such as a reduction in neuronal proliferation, have been observed acutely: For instance, within days after 0.2–1 Gy of 300 MeV/n ^28^Si irradiation [30], though the majority of cognitive and associated physiological outcomes occur at later time points. Overall, cognitive and behavioral deficits have been reported as early as 48 h post exposure to 0.3 and 1 Gy of 1 GeV/n ^56^Fe [29], and as late as 1 year after exposure to 0.05 and 0.3 Gy of 400 MeV/n ^4^He [8]. Thus, the outcomes of HZE ion exposure may eventually overlap with natural aging processes and potentially exacerbate them, since radiation responses resemble senescence at the molecular and cellular level [31]. In addition, age is an important factor by itself, because it changes baseline CNS functions by increasing neuroinflammation and ROS production [43], thus it could be expected to alter the responses to radiation as well. Indeed, older rats respond to the same dose of 1.5 Gy of 1 GeV/n ^56^Fe ions by developing worse cellular and behavioral impairments [26]. This is an important consideration, because most research to date has been performed in young animals (~2 months of age), while astronauts typically are middle age when they begin their careers, which corresponds to at least 6 months of age in rodents. It also expands the research focus to include post-flight adaptation in humans and animal models and extends the period for application of therapeutic countermeasures.

The interpretation of the experimental results is further complicated by the limitations of simulating GCRs on ground: Most animal experiments use a single dose of protons, ^4^He, or HZE particles of which ^16^O, ^28^Si, ^48^Ti and ^56^Fe ions are the most common. However, each particle may have different relative biological efficiency in the CNS, and their combination may lead to additional novel outcomes based on synergistic interaction. Some of the cellular effects caused by different particles are comparable, such as methylation changes induced by protons and ^56^Fe ions [12], and microglial activation caused by protons, ^16^O, ^48^Ti and ^4^He ions [8,13]. However, other studies indicate that rodent cognitive deficits are significantly more pronounced when caused by heavy ions or ^4^He compared to protons alone [44]. These issues are partially mitigated by recently developed experimental setups for consecutive exposures to multiple ions in GCR simulations [45] and by chronic low-dose particle irradiation [46]. However, the drawback of GCR simulations is that they consist of sequential irradiations by multiple ions, which may have different effects based on the order of irradiation. For example, 0.1 Gy of 150 MeV/n proton exposure 24 h prior to 0.5 Gy 600 MeV/n ^56^Fe irradiation leads to partially, but not completely, distinct behavioral effects and cytokine secretion patterns from either protons or ^56^Fe ions alone [15]. Therefore, to achieve accurate comparisons, more studies are needed to relate the response to unique ions with their combination. Specifically, measuring the latency of cellular effect at very low fluence may bring up clues for the significance of synergism the order of ions. Such information would reduce uncertainty when evaluating the relative biological effectiveness (RBE) of the combined simulated GCRs compared to their individual components on the CNS.

Furthermore, in order to consider how long-term space travel can alter CNS functions, it is necessary to investigate the potential interactions between radiation, microgravity and social isolation. Simulated microgravity using the hindlimb unloading model is sufficient to increase oxidative stress [47] and alter brain functions by reorganizing the map of the unloaded limb in the motor cortex [48]. Interestingly, it has been shown to increase dendritic spine formation in rats [49], which is the opposite of the effects of HZE ions. On the other hand, hindlimb unloading leads to glial activation in the spinal cord [50], which is similar to HZE irradiation outcomes. Similarly, spaceflight to the ISS induces oxidative stress and changes in inflammatory signaling [51]. In addition, radiation may act synergistically with other factors such as high CO_2_ levels on the spacecraft [52], because both induce oxidative stress responses. Thus, the combination of gravitational changes, space environment and GCR exposure may have complex synergistic effects that need to be studied.

Astronauts also experience social isolation, which in experimental models has been shown to impair synapse functions in the hippocampus [53] and social recognition memory [54]. The effects of social isolation have previously been studied in combination with another major stressor, in this case, stroke. Mouse isolation after stroke has been demonstrated to increase depressive behavior and reduce brain-derived neurotrophic factor [55]. Similarly, in spaceflight the additional variable of social stress may further exacerbate or attenuate the cognitive and neurophysiological effects of radiation and microgravity.

Considering the astronaut return to the Earth, at the moment it remains inconclusive whether and how particle radiation could exacerbate the outcomes of neurodegenerative diseases. For example, in a mouse model of Alzheimer’s disease, 0.1–1 Gy of 1 GeV/n ^56^Fe irradiation or 0.1–1 Gy of 150 MeV/n proton irradiation exacerbate the deposition of amyloid-beta and worsens the electrophysiological dysfunction of synaptic connections [19,21]. The interactions between radiation and traumatic brain injury are even more complex: 0.5 Gy of 600 MeV/n ^56^Fe irradiation has been shown to be neuroprotective against acute CNS trauma, possibly by preconditioning the neurons to adaptation against repeated insults [16], though in contrast, high doses of gamma radiation are actually detrimental to traumatic brain injury [56].

Finally, these reports have primarily used male animals, a potential confounder as more and more studies are reporting sex-dimorphic responses to cognitive alterations. Forty percent of the most recent class of astronauts is female, therefore it is important to understand how GCR exposure impacts both sexes. A recent study has shown female mice to be significantly more resistant to CNS effects of low dose ionizing radiation compared to male. This sex-specific resistance in cognitive and behavioral outcomes is also paralleled by the cellular changes in hippocampal synapse loss and microgliosis that are not observed in females [6]. Long-term responses to ^28^Si irradiation showed a worse reduction in new neuron survival in male than in female mice [30], though one previous study has indicated the opposite effect of higher doses of ^56^Fe irradiation: Impaired memory responses in female mice, but improved in male mice [57]. In combination, these results emphasize the necessity of considering sex, age and other health conditions in experimental design and applicability to human spaceflight, and encourage the investigation of sex-specific mechanisms that may account for the difference in radiation sensitivity. One potential focus of the future studies using female mice could be to investigate how the estrus cycle affects the response of the CNS by tracking the stage of mice’s cycle at the time of exposure. 

## 5. Novel Approaches for Investigating CNS Responses of Humans to Simulated Cosmic Radiation and Countermeasure Development

In addition to increasing our understanding of CNS risks during exposure to the deep space environment, more research is needed to understand how to translate the experimental results to human spaceflight missions. Recent research directions include identifying peripheral biomarkers for predicting the severity of CNS impairments, genetic diversity of radiation sensitivity from cellular to organism level, and evaluating radiation responses in human tissue. Most biomarkers of radiation responses have been based either on the molecular composition of the serum in response to whole-body radiation damage [58] or immune cell populations correlating with CNS deficits [22]. The serum signature of radiation injury in the CNS remains to be characterized, possibly by adapting already existing molecular biomarkers for brain injury [59] or neurodegeneration [60], based on the overlapping mechanisms of oxidative stress and neuroinflammation. An alternative approach would be to focus on mechanisms governing individual cell responses to radiation injury: Recent studies report high variability between neurons [61], microglia [62] and astrocytes [63] at the level of gene expression in health and disease, which suggests a potentially equal intercellular variability in radiation responses that could lead to cell-specific targets for countermeasures. 

Designing human CNS equivalents to study physiological responses to perturbations (including space radiation) is an active field of research. So far, the most promising approaches could be classified into three groups: Induced pluripotent stem cells to replicate the individual genetic variability in vitro in both neurons and glia [64,65], cultured spherical 3D brain organoids [66,67], and high-throughput multicellular organs-on-a-chip replicating 3D brain structure and blood-brain barrier [68,69,70]. These novel technologies might be adapted to investigate exposure to space radiation, and especially to combined exposures that address multiple space-relevant stressors. 

The development of therapeutic countermeasures to protect CNS from GCR exposure is poorly studied due to lack of full understanding of the GCR exposure to the brain. A few studies have been able to demonstrate that targeting inflammation and/or ROS pathways could provide a valuable countermeasure to evaluate. Limiting ROS production and improving mitochondrial function has been shown to rescue hippocampal neurogenesis and reduce neuroinflammation and cognitive impairments [14,17,41]. However, most of these studies rely on transgenic animal models, which have limited therapeutic applicability. This obstacle has recently been overcome by using small molecules to reduce neuroinflammation by temporarily depleting the microglia [13] after irradiation. Other anti-inflammatory treatments for GCRs could be developed based on the mechanisms used to reduce inflammation after gamma radiation, e.g., blockade of chemotactic signaling to prevent myeloid cell migration and differentiation into pro-inflammatory phenotype.

Other novel approaches not yet studied in the context of CNS responses to space radiation might include the blood-brain barrier, which exacerbates neuroinflammation by allowing peripheral immune cell influx into the CNS, or astrocytes, which regulate neuronal health, neuroinflammation and blood-brain barrier permeability [71]. Microglial functions, especially microglia-mediated synapse loss, could also be altered by targeting the complement cascade [34]. Finally, one of the overarching mechanisms that governs astrocytic responses to injury, microglia and complement functions, and neuronal survival is a major regulatory cytokine transforming growth factor-beta1 [72,73], which also mediates rodent responses to spaceflight and is upregulated by very low doses of radiation [74], thus it could serve as a suitable target for countermeasure development.

The most cutting-edge methods that may be utilized to improve CNS resistance to GCRs include altering gene expression using microRNAs [75] or CRISPR-based RNA editing [76] in peripheral immune cells, if the blood-brain barrier is sufficiently disrupted to allow them to penetrate into the brain. Alternatively, peripherally injected pharmaceuticals could be carried through the blood-brain barrier by nanoparticles [77]. Finally, there is emerging evidence that normal astrocyte and microglial functions and neuronal health considerably depend on the metabolites produced by the gut microbiome [78,79,80,81]. Thus, the microbiome could be targeted to improve CNS resistance to radiation in a comparatively non-invasive manner.

## 6. Conclusions

In summary, the main risks of cosmic radiation for the CNS include oxidative stress and neuroinflammation that lead to neuronal damage and cognitive deficits. In order to improve diagnosis and treatment of possible CNS disorders in human spaceflight, it will be necessary to develop more accurate prediction models for radiation responses, including age and sex as major epidemiological factors, and supplement the animal studies with cellular and tissue equivalents with the focus on human cellular responses. These models could then be utilized to build on the current knowledge on limiting oxidative stress and reducing neuroinflammation by using novel methods and mechanisms for targeting the immune response in the brain or the rest of the body. Finally, when accounting for the effect of prolonged spaceflight and deep space exploration on the CNS it is important to determine the possible compounding effects of GCR with other significant stressors such as microgravity, space environment like CO_2_ levels and social isolation.

## Figures and Tables

**Table 1:** Summary of central nervous system (CNS) cellular and tissue damage in response to HZE ion irradiation.

Targets	Damage	Particle and Dose	Time After Exposure	References
**Neuronal**	Dendritic, axonal and synaptic degeneration	600 MeV/n ^16^O: 0.05, 0.3 Gy 600 MeV/n ^48^Ti: 0.05, 0.3 Gy 600 MeV/n ^56^Fe: 4 Gy GCR: 0.5 Gy	3 months 3 months 1, 6, 12 months 4 months	[6,8,32]
	Neuronal excitability changes	150 MeV/n protons: 1 Gy	3 months	[33]
	Neuronal proliferation deficits	300 MeV/n: 0.2, 1 Gy 1 GeV/n ^56^Fe: 0.3, 1 Gy	24 h 48 h	[29,30]
	Neuronal death	1 GeV/n ^56^Fe: 1.6 Gy 500 MeV/n ^56^Fe: 1.5 Gy	12 months 1 month	
**Glial**	Astrocyte activation	1 GeV/n ^56^Fe: 1.6 Gy 600 MeV/n ^56^Fe: 4 Gy	12 months 1, 6, 12 months	[31,32]
	Microglial activation	400 MeV/n ^4^He: 0.05, 0.3 Gy 400 MeV/n ^4^He: 0.15, 0.5 Gy 600 MeV/n ^16^O: 0.05, 0.3 Gy 600 MeV/n ^48^Ti: 0.05, 0.3 Gy GCR	12 months 3 months 15, 27 weeks 15, 27 weeks 4 months	[6,8,13,20]
**Tissue-level**	Oxidative stress	150 MeV/n protons, 0.5, 2 Gy 1 GeV/n ^56^Fe, 1.6 Gy 500 MeV/n ^56^Fe, 1.5 Gy	1 month 2, 12 months 1 month	[14,17,31]
